# Clinical impact of primary tumor sidedness and sex on unresectable post-recurrence survival in resected pathological stage II-III colorectal cancers: a nationwide multicenter retrospective study

**DOI:** 10.1186/s12885-022-09615-z

**Published:** 2022-05-02

**Authors:** Shinya Abe, Kazushige Kawai, Hiroaki Nozawa, Kazuhito Sasaki, Koji Murono, Shigenobu Emoto, Tsuyoshi Ozawa, Yuichiro Yokoyama, Yuzo Nagai, Hiroyuki Anzai, Hirofumi Sonoda, Shinichi Yamauchi, Kenichi Sugihara, Soichiro Ishihara

**Affiliations:** 1 Department of Surgical Oncology, Graduate School of Medicine, The University of Tokyo, 7-3-1 Hongo, Bunkyo-ku, Tokyo, 113-0033 Japan; 2 Department of Surgical Oncology, Graduate School of Medicine, The Tokyo Medical and Dental University, Bunkyo-ku, Tokyo, Japan

**Keywords:** Primary tumor location, Prognostic marker, Colorectal cancer, Chemoradiotherapy, Outcome

## Abstract

**Background:**

Several studies have demonstrated that right-sided tumors have poorer prognosis than left-sided tumors in patients with unresectable colorectal cancer (CRC). The predictive ability of the tumor sidedness in CRC treated with chemotherapy in each sex is unclear.

**Methods:**

Subjects were 964 unresectable recurrent patients treated with chemotherapy with stage II-III CRC after curative resection between 2004 and 2012. Post-recurrence cancer-specific survival (CSS) for each sex was examined.

**Results:**

Patients were 603 males (222 right-side tumors (cecum to transverse colon) and 381 left-sided tumors (descending colon to rectum)), and 361 females (167 right-side tumors and 194 left-sided tumors). Right-sided tumors developed peritoneal recurrences in males and females. Left-sided tumors were associated with locoregional recurrences in males and with lung recurrences in females. Right-sided tumors were associated with shorter post-recurrence CSS in both sexes. In males, multivariate analyses showed that right-sided tumors were associated with shorter post-recurrence CSS (HR: 1.53, *P* < 0.0001) together with the presence of regional lymph node metastasis histopathological type of other than differentiated adenocarcinoma, the recurrence of liver only, the recurrence of peritoneal dissemination only, and relapse-free interval less than one-year. In females, multivariate analyses showed that right-sided tumors were associated with shorter post-recurrence CSS (HR: 1.50, *P* = 0.0019) together with advanced depth of invasion, the presence of regional lymph node metastasis, and recurrence of liver only.

**Conclusions:**

Primary tumor sidedness in both sexes in unresectable recurrent CRC patients treated with chemotherapy may have prognostic implications for post-recurrence CSS.

**Supplementary Information:**

The online version contains supplementary material available at 10.1186/s12885-022-09615-z.

## Introduction

Colorectal cancer (CRC) is one of the most common malignancies and most frequent causes of cancer-related death worldwide [1]. In order to decide personalized treatment or postoperative follow-up strategies, suitable predictive and prognostic indicators are necessary.

Recently, primary tumor sidedness is being considered as a prognostic and predictive marker. Previous studies demonstrated that stage IV patients with right-sided tumors had worse prognosis due to peritoneal dissemination, unresectable liver metastases, and unresectable synchronous metastasis [2–4]. Primary tumor sidedness stratifies outcomes in metastatic colorectal cancers; however, its prognostic value is controversial in nonmetastatic colorectal cancers [5–8]. We previously reported that patients with right-sided tumor have shorter cancer-specific survival after recurrence in nonmetastatic colon cancer [9]. Malakorn et al. reported that right-sided colon cancer was more likely to develop multi-focal site recurrence among 71 patients that developed recurrence, and had worse survival after recurrence [10]. However, the recurrence pattern has not been fully investigated in a large-scale study.

Currently, primary tumor location is a treatment strategy criterion for unresectable CRC patients in many countries including Japan. Guidelines, such as the European Society for Medical Oncology, National Comprehensive Cancer Network, and Japanese Society for Cancer of the Colon and Rectum guidelines [11–13], recommend that an anti-EGFR antibody is used in left-sided but not right-sided tumors for KRAS and BRAF wild patients. These recommendations are based on evidence from both individual trial findings, such as the CRYSTAL, PRIME, PEAK, FIRE-3, CALGB 80405, and 20,050,181 trials, and pooled data analyses [14–21]. Thus, KRAS and BRAF mutation and tumor sidedness are useful factors for deciding treatment strategy. Moreover, microsatellite instability (MSI) and TP53 mutation are also predictive markers for chemotherapeutic effect, and the prevalence of MSI-high and TP53 mutation are reported to differ by tumor location and sex [22–24]. The present study aimed to clarify the relationship between tumor sidedness in each sex and unresectable post-recurrence survival in patients with colorectal cancer treated with chemotherapy.

## Materials and methods

### Study population

A total of 22,638 patients’ data were collected by The Japanese Study Group for Follow-Up of Colorectal Cancer. All patients had pathologically diagnosed stage II-III colorectal cancer and underwent curative surgery at 24 Japanese referral hospitals between January 2004 and December 2012. Inclusion criteria were recurrent disease after curative resection of the primary tumor and received chemotherapy, which was not palliative, without resection of recurrent tumor. Exclusion criteria included: histology other than adenocarcinoma, hereditary disease, inflammatory bowel disease, multiple colorectal cancers, multiple primary tumors, insufficient clinical and pathological information, unknown stage information, and unknown follow-up information. This study was approved by the Central Institutional Review Board (Tokyo Medical and Dental University [No. M2017–268]) and the Ethics Review Board of each institution, including the ethics committees of the University of Tokyo (No.3252- [13]).

### Patient follow-up

Patients underwent postoperative surveillance at 3-month intervals after surgery according to the surveillance protocol, as described previously [9, 13]. Regular tumor marker assessment of serum carcinoembryonic antigen and carbohydrate antigen 19–9 (every 3 months), computed tomography scans (every 6 months), and colonoscopy examinations (every year) were performed for 5 years postoperatively. Follow-up data were recorded until the study cutoff date of January 2019. Patient data were extracted from the prospectively generated database of each institute and analyzed retrospectively.

### Data selection

Patients were divided into two groups according to tumor location (right-sided tumor or left-sided tumor). A right-sided tumor was defined as a tumor of the cecum, ascending colon, or transverse colon. A left-sided tumor was defined as a tumor of the descending colon, sigmoid colon, or rectum. The following clinical factors were assessed using medical records: sex, age, treatment year, primary tumor location, histological type, pathological TNM classification, stage (American Joint Committee on Cancer classification), and the presence of adjuvant chemotherapy. The site of recurrence and treatment strategy was also included for patients with recurrence.

### Statistical analysis

Clinicopathological features were analyzed using the Mann-Whitney U-test for continuous variables or the chi-square test for categorical variables. Overall survival (OS) was determined using the records from surgery date to death or last follow-up. Post-recurrence cancer-specific survival was defined using the records from recurrence after radical surgery for the primary tumor to cancer-specific death or last follow-up. Post-recurrence cancer-specific survival was analyzed using Kaplan-Meier survival curves and log-rank tests. Predictive factors for post-recurrence cancer-specific survival were assessed using: primary tumor location (left vs. right), age (< 75 years vs. ≥ 75 years), depth of invasion (T1–3 vs. T4), lymph node metastasis (absent vs. present), adjuvant therapy (present vs. absent), histopathological type (differentiated vs. others), and relapse-free interval (< 1 year vs. ≥ 1 year). Variables with a *P*-value of < 0.1 in the univariate analyses were further evaluated using the Cox proportional hazards model to determine the predictive factors. Data analyses were performed using JMP Pro 16.0 software (SAS Institute Inc., Cary, NC, USA). A *P* value < 0.05 was considered significant.

## Results

### Patient characteristics

**Fig. 1:**
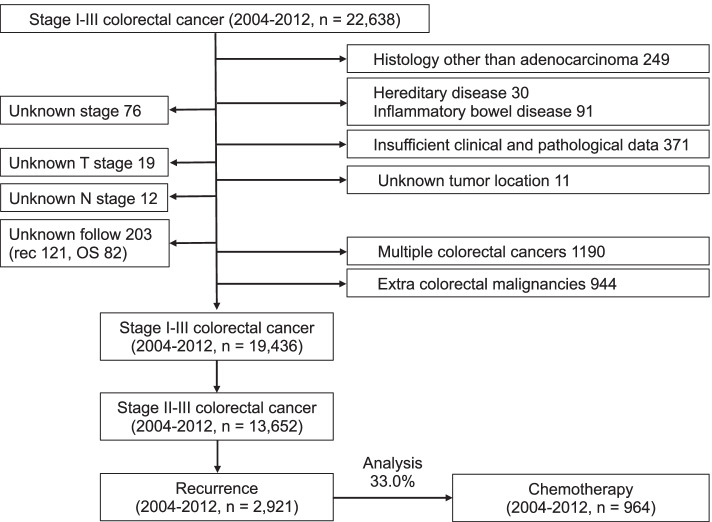
Study cohort selection process. The Japanese Study Group for Follow-Up of Colorectal Cancer collected clinicopathological data from 22,638 patients with pathological stage II-III colorectal cancer who underwent radical surgery between January 2004 and December 2012 at 24 Japanese referral hospitals. A total of 964 patients with unresectable recurrent colorectal cancer treated with chemotherapy were included in the final study population. Patients treated with palliative chemotherapy were excluded

The present study cohort comprised 964 patients with colorectal cancer, including 603 male patients and 361 female patients (Fig. 1). In the males, 222 (23.0%) patients had right-sided tumor and 381 (39.5%) patients had left-sided tumor, and in the females, 167 (17.3%) patients had right-sided tumor and 194 (20.2%) patients had left-sided tumor. Patient characteristics are summarized in Table 1. In all cohorts, the median of the recurrence-free interval was 1.41 years. Recurrence sites were: liver only (*n* = 240, 24.9%), lung only (*n* = 267, 27.7%), peritoneum only (*n* = 123, 12.8%), extra-regional lymph node only (*n* = 140 patients, 14.5%), locoregional only (*n* = 120, 12.4%), anastomotic site only (*n* = 8, 0.8%), bone only (*n* = 19, 2.0%), and multiple organ recurrences (*n* = 44, 4.6%). The median of the recurrence-free interval was 0.58 (interquartile range [IQR]: 0.41–1.17) years in liver only, 1.17 (IQR: 0.67–1.92) years in lung only, 1.08 (IQR: 0.83–1.75) years in peritoneum only, 1.33 (IQR: 0.69–2.48) years in extra-regional lymph node only, 1.25 (IQR: 0.67–2.17) years in locoregional only, 1.41 years in anastomotic site only, and 0.75 (IQR: 0.33–1.67) years in bone only. These intervals were significantly different in each organ (*P* < 0.0001). On the other hand, there were no differences in the median intervals between male and female (1.0 and 1.0 years, respectively, *P* = 0.2440), right-sided and left-sided tumor (1.0 and 1.0 years, respectively, *P* = 0.0819), and stages II and III (1.08 and 1.0 years, respectively, *P* = 0.1922).

**Table 1:** Clinicopathological features according to sex

			Male			Female		
Variables		All	Right (*n* = 222)	Left (*n* = 381)	*P* value	Right (*n* = 167)	Left (*n* = 194)	*P* value
Age (years)	(mean ± SD)	64.9 ± 11.1	65.7 ± 11.2	64.0 ± 10.6	0.0670	66.7 ± 11.0	64.1 ± 11.6	0.0277
Depth of invasion	T1-T3	570 (59.1%)	122 (54.9%)	256 (67.2%)	0.0028	77 (46.1%)	115 (59.3%)	0.0123
	T4	394 (40.9%)	100 (45.1%)	125 (32.8%)		90 (53.9%)	79 (40.7%)	
Lymph node metastasis	Absent	278 (28.8%)	69 (31.1%)	119 (31.2%)	0.9689	41 (24.6%)	49 (25.3%)	0.877
	Present	686 (71.2%)	153 (68.9%)	262 (68.8%)		126 (75.4%)	145 (74.7%)	
Adjuvant therapy	Absent	379 (39.3%)	91 (41.0%)	154 (40.4%)	0.6138	64 (38.3%)	70 (36.1%)	0.8811
	Present	561 (58.2%)	126 (56.8%)	213 (55.9%)		101 (60.5%)	121 (62.4%)	
	Unknown	24 (2.5%)	5 (2.2%)	14 (3.7%)		2 (1.2%)	3 (1.6%)	
Histopathological type	Differentiated	875 (90.8%)	197 (88.7%)	346 (90.8%)	0.4151	149 (89.2%)	183 (94.3%)	0.0749
	Others	89 (9.2%)	25 (11.3%)	35 (9.2%)		18 (10.8%)	11 (5.7%)	
Recurrence-free interval (years)	(mean ± SD)	1.41 ± 1.25	1.36 ± 1.25	1.51 ± 1.39	0.1873	1.29 ± 1.13	1.41 ± 1.06	0.3251
Recurrence pattern								
Single organ								
Liver	Present	240 (24.9%)	64 (30.0%)	100 (27.3%)	0.4717	40 (25.2%)	36 (19.3%)	0.1867
Lung	Present	267 (27.7%)	54 (25.4%)	104 (28.3%)	0.4347	36 (22.6%)	73 (39.0%)	0.0010
Peritoneal dissemination	Present	123 (12.8%)	40 (18.8%)	29 (7.9%)	0.0001	36 (22.6%)	18 (9.6%)	0.0009
Extra-regional lymph node	Present	140 (14.5%)	26 (12.2%)	50 (13.6%)	0.6243	27 (17.0%)	37 (19.8%)	0.5022
Locoregional	Present	120 (12.4%)	20 (9.4%)	68 (18.5%)	0.0023	15 (9.4%)	17 (9.1%)	0.9126
Anastomotic sight	Present	8 (0.8%)	1 (0.5%)	5 (1.3%)	0.2746	1 (0.6%)	1 (0.5%)	0.9085
Bone	Present	19 (2.0%)	3 (1.4%)	12 (3.3%)	0.1542	0	4 (2.1%)	0.0259
Multiple organs	Present	44 (4.6%)	12 (5.4%)	17 (4.7%)	0.6042	8 (4.8%)	7 (3.6%)	0.5753

*SD* Standard deviation

Male patients showed significant differences in depth of invasion, recurrence in peritoneal dissemination only, and locoregional only between the groups. Males with right-sided tumor were more likely to have T4 cancer and have recurred with peritoneal dissemination only, and less likely to have recurred with locoregional only.

On the other hand, significant differences in female patients were observed in age, depth of invasion, recurrence in lung metastasis only, peritoneal dissemination only, and bone metastasis only between the groups. Females with right-sided tumor were more likely to be older, have T4 cancer, and have recurred with peritoneal dissemination only, and less likely to have recurred with lung only and bone only metastasis.

### Post-recurrence cancer-specific survival

**Fig. 2:**
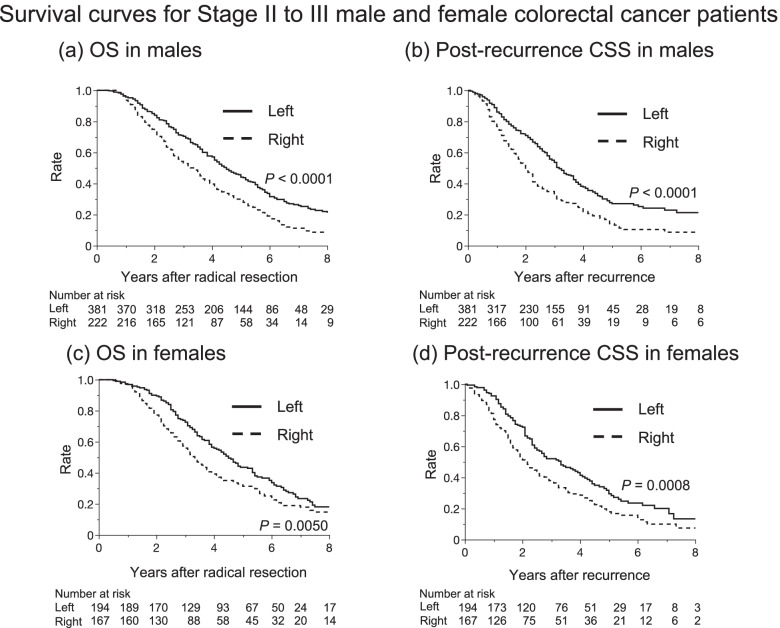
Correlation among left-sided and right-sided tumors and overall survival (OS) and post-recurrence cancer-specific survival (CSS) according to sex. **a** OS curves in males stratified according to tumor sidedness (5-year OS for left-sided tumors 45.8%, right-sided tumors 30.0%). **b** Post-recurrence CSS curves in males stratified according to tumor sidedness (5-year post-recurrence CSS for left-sided tumors 27.7%, right-sided tumors 14.2%). **c** OS curves in females stratified according to tumor sidedness (5-year OS for left-sided tumors 43.6%, right-sided tumors 31.8%). **d** Post-recurrence CSS curves in females stratified according to tumor sidedness (5-year post-recurrence CSS for left-sided tumors 31.8%, right-sided tumors 17.8%)

The median follow-up period was 43.0 months, and the 5-year overall survival (OS) and post-recurrence cancer-specific survival (CSS) were 38.3 and 19.4%, respectively (data not shown). The OS and post-recurrence CSS of the entire cohort, stratified by tumor location. The 5-year OS of patients with left-sided tumor and right-sided tumor were 45.1 and 31.2%, respectively (*P* < 0.0001). Moreover, the 5-year post-recurrence CSS of patients with left-sided tumor and right-sided tumor were 29.1 and 15.8%, respectively (*P* < 0.0001). Furthermore, we investigated the prognostic capability of sex in these survival curves. Tumor location stratified OS and post-recurrence CSS curves in male and female patients. The survival curves were more clearly stratified in male patients than in female patients (Fig. 2).

### Post-recurrent cancer-specific survival stratified by tumor location according to recurrence site

**Fig. 3:**
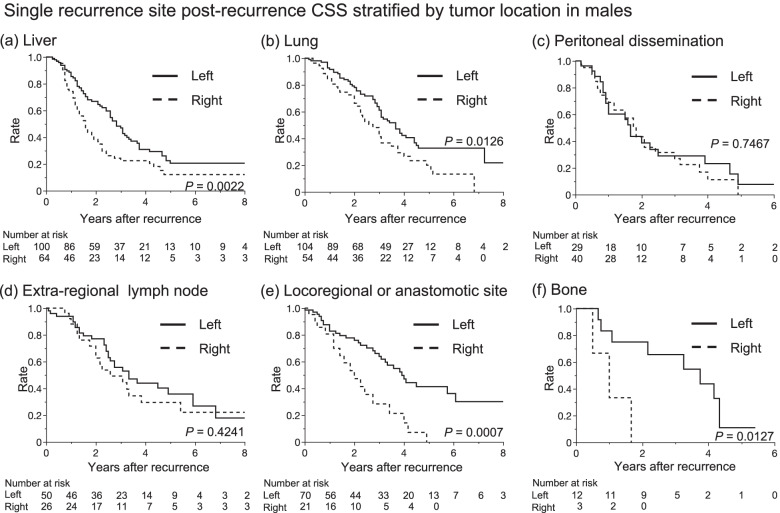
Correlation among left-sided and right-sided tumors and post-recurrence cancer-specific survival (CSS) in males according to single organ recurrence in: (**a**) liver, (**b**) lung, (**c**) peritoneum, (**d**) extra-regional lymph node, (**f**) locoregional or anastomotic site, and (**g**) bone metastasis. Kaplan-Meier curves in the liver (**a**), lung (**b**), locoregional or anastomotic site (**e**), and bone (**f**) metastasis were significantly stratified according to tumor sidedness. Tumor-sidedness did not stratify other Kaplan-Meier curves in peritoneal (**c**) and extra-regional lymph node (**d**) metastasis

**Fig. 4:**
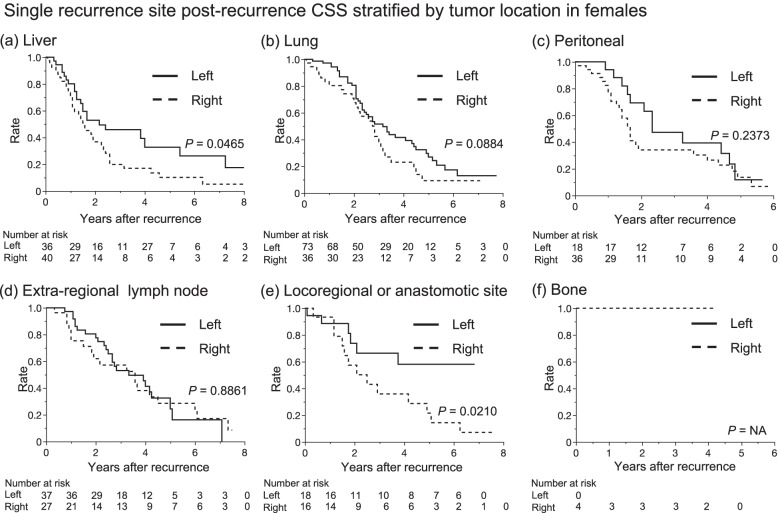
Correlation among left-sided and right-sided and post-recurrence cancer-specific survival in females according to single organ recurrence in (**a**) liver, (**b**) lung, (**c**) peritoneum, (**d**) extra-regional lymph node, (**f**) locoregional or anastomotic site, and (**g**) bone metastasis. According to tumor sidedness, Kaplan-Meier curves in the liver (**a**) and locoregional or anastomotic site (**e**) metastasis were significantly stratified. Tumor-sidedness did not stratify other Kaplan-Meier curves in the lung (**b**), peritoneum (**c**), extra-regional lymph node (**d**), and bone (**f**) metastasis

Metastatic organ site is a potent prognostic factor; thus, we focused on the differences in organ-specific survival rate in post-recurrent CCS in each sex. The 3-year post-recurrence CSS rates of male patients among those with left-sided tumor and right-sided tumor were 48.3 and 24.4% in liver only (*P* = 0.0022), 63.8 and 48.2% in lung only (*P* = 0.0126), 29.0 and 31.5% in peritoneal dissemination only (*P* = 0.7467), 55.7 and 49.3% in extra-regional lymph node only (*P* = 0.4241), 65.6 and 0% in bone only (*P* = 0.0127), and 66.1 and 28.4% in either locoregional or anastomotic only (*P* = 0.0007), respectively (Fig. 3). On the other hand, the 3-year post-recurrence CSS rates of female patients among those with left-sided tumor and right-sided tumor were 45.9 and 19.8% in liver only (*P* = 0.0465), 51.6 and 42.3% in lung only (*P* = 0.0884), 47.3 and 34.2% in peritoneal dissemination only (*P* = 0.2373), 53.1 and 57.2% in extra-regional lymph node only (*P* = 0.8861), 100% and not applicable (no patient) in bone only (*P* = 0.0210), and 66.4 and 35.9% in either locoregional or anastomotic only (*P* = 0.0210), respectively (Fig. 4). In multiple organ recurrence patients, tumor location did not stratify significantly in post-recurrence CSS; the 3-year post-recurrence CSS rates among those with left-sided tumor and right-sided tumor in male and female were 39.7 and 33.3% (*P* = 0.9950) and 51.4 and 46.9% (*P* = 0.8118), respectively.

### Factors affecting post-recurrence cancer-specific survival in each sex

Tables 2 and 3 show the results of univariate and multivariate analyses of factors affecting post-recurrence CSS in male and female patients. Among male patients, right-sided tumor was an independent risk factor (hazard ratio, 1.53; 95% confidence interval (CI) 1.31–1.80; *P* < 0.0001) together with the presence of regional lymph node metastasis (HR: 1.47; 95% CI 1.23–1.76; *P* < 0.0001), histopathological type of other than differentiated adenocarcinoma (HR: 1.55; 95% CI 1.18–2.03; *P* = 0.0014), the recurrence of liver only (HR: 1.33; 95% CI 1.09–1.63; *P* = 0.0059), the recurrence of peritoneal dissemination only (HR: 1.52; 95% CI 1.19–1.94; *P* = 0.0007), and relapse-free interval less than one-year (HR: 1.30; 95% CI 1.10–1.54; *P* = 0.0019). On the other hand, among female patients, multivariate analyses showed that right-sided tumors were associated with shorter post-recurrence CSS (HR: 1.50; 95% CI 1.16–1.94; *P* = 0.0019) together with advanced depth of invasion (HR: 1.61; 95% CI 1.24–2.09; *P* = 0.0004), the presence of regional lymph node metastasis (HR: 1.38; 95% CI 1.02–1.87; *P* = 0.0366), and recurrence of liver only (HR: 1.57; 95% CI 1.16–2.13; *P* = 0.0036).

**Table 2:** Univariate and multivariate analyses of prognostic variables for post-recurrence cancer-specific survival in males

		Univariate analysis			Multivariate analysis		
		HR	95% CI	*P* value	HR	95% CI	*P* value
Primary tumor location	Left vs. Right	1.72	1.41–2.11	< 0.0001	1.53	1.31–1.80	< 0.0001
Age (years)	< 75 vs. ≥ 75	1.49	1.10–1.81	0.0076	1.19	0.98–1.46	0.0853
Depth of invasion	T1–3 vs. T4	1.08	0.86–1.33	0.4806			
Regional lymph node metastasis	Absent vs. Present	1.44	1.16–1.81	0.0012	1.47	1.23–1.76	< 0.0001
Histopathological type	Differentiated vs. Others	1.41	1.09–1.84	0.0100	1.55	1.18–2.03	0.0014
Recurrence of liver only	Absent vs. Present	1.36	1.14–1.61	0.0005	1.33	1.09–1.63	0.0059
Recurrence of lung only	Absent vs. Present	1.25	1.05–1.49	0.0111	0.96	0.79–1.17	0.6682
Recurrence of peritoneal dissemination only	Absent vs. Present	1.55	1.24–1.93	0.0001	1.52	1.19–1.94	0.0007
Recurrence of extra-regional lymph node only	Absent vs. Present	0.85	0.69–1.05	0.1409			
Recurrence of bone only	Absent vs. Present	1.15	0.70–1.88	0.5920			
Recurrence of locoregional only	Absent vs. Present	0.80	0.62–1.03	0.0786			
Recurrence of anastomosis only	Absent vs. Present	0.97	0.46–2.05	0.9448			
Number of recurrent organs	Single vs Multiple	1.38	0.97–1.96	0.0716			
Relapse-free interval (years)	≥ 1.0 vs. < 1.0	1.42	1.21–1.66	< 0.0001	1.30	1.10–1.54	0.0019

*HR* Hazard ratio, *CI* Confidential interval

**Table 3:** Univariate and multivariate analyses of prognostic variables for post-recurrence cancer-specific survival in females

		Univariate analysis			Multivariate analysis		
		HR	95% CI	*P* value	HR	95% CI	*P* value
Primary tumor location	Left vs. Right	1.54	1.19–1.98	0.0009	1.50	1.16–1.94	0.0019
Age (years)	< 75 vs. ≥ 75	0.40	0.83–1.60	0.3970			
Depth of invasion	T1–3 vs. T4	1.60	1.23–2.07	0.0004	1.61	1.24–2.09	0.0004
Regional lymph node metastasis	Absent vs. Present	1.38	1.02–1.87	0.0342	1.38	1.02–1.87	0.0366
Histopathological type	Differentiated vs. Others	1.16	0.74–1.84	0.5155			
Recurrence of liver only	Absent vs. Present	1.64	1.22–2.19	0.0009	1.57	1.16–2.13	0.0036
Recurrence of lung only	Absent vs. Present	0.88	0.67–1.15	0.3463			
Recurrence of peritoneal dissemination only	Absent vs. Present	1.32	0.94–1.86	0.1099			
Recurrence of extra-regional lymph node only	Absent vs. Present	0.86	0.62–1.18	0.3453			
Recurrence of bone only	Absent vs. Present	0.50	0.07–3.55	0.4854			
Recurrence of locoregional only	Absent vs. Present	0.70	0.44–1.12	0.1392			
Recurrence of anastomosis only	Absent vs. Present	0.88	0.22–3.57	0.8632			
Number of recurrent organs	Single vs Multiple	1.15	0.57–2.33	0.6940			
Relapse-free interval (years)	≥ 1.0 vs. < 1.0	1.32	1.02–1.70	0.0354	1.16	0.89–1.51	0.2699

*HR* Hazard ratio, *CI* Confidential interval

## Discussion

The present study analyzed post-recurrence CSS in pathologically diagnosed stage II–III colorectal cancer, focused on sex and recurrence sites. Multivariable analysis showed that post-recurrence CSS was significantly lower in those with right-sided tumor among both male and female patients. Thus, primary tumor location should be considered for treatment strategy and clinical practice, regardless of sex.

In the present study, the recurrence-free interval in each organ was different, the intervals of liver metastasis and anastomotic site recurrence were shorter than other metastatic sites. The recurrence-free interval in each organ in the present study was shorter than a previous study [25]. An unresectable recurrent status may be more aggressive than a resectable recurrent status because short recurrence-free survival, especially shorter than 1 year, was demonstrated to be a negative predictive factor for several malignancies including colorectal, rectal, and gastric cancer [25–27].

In the present study, CSS after recurrence depended on recurrent organs, which was in line with a previous study [28]. However, the recurrence pattern among male and female patients has not been fully investigated in a large cohort. A previous study demonstrated that right-sided tumors had more favorable peritoneal recurrence compared with left-sided tumors [9, 29], this study also found the same trend in both sexes. Locoregional recurrence was observed more frequently in left-sided tumors in males. Anastomotic leakage after colorectal surgery increases mortality, and males and rectal cancer have been reported to be risk factors due to the smaller pelvis and more substantial muscular wall in males in a large cohort study, which is supportive to our results [30]. The recurrence ratio in lung was higher in left-sided tumors among females, which is in line with previous large cohort studies [31, 32], and being female was the only adverse prognostic indicator for the development of lung metastasis [32].

Several factors have been proposed to affect survival between right-sided and left-sided tumors. First, the proximal colon from the cecum to two-thirds of the oral side of the transverse colon (right-sided) is derived from the embryonic midgut, and the distal third of the transverse colon to the rectum (left-sided) is derived from the embryonic hind-gut [21]. Second, their function is different as the right-sided colon mainly absorbs water, whereas the left-sided colon facilitates the passage of bowel contents. Third, there was a difference in gut microbiota composition. Prevotella, Pyramido-bacterium, Selenomonas, and Peptostreptococcus were identified at relatively higher abundance in right-sided CRC, whereas Fusobacterium, Escherichia/Shigella, and Leptotrichia were relatively abundant in left-sided CRC [33]. *Fusobacterium nucleatum* was reported to promote chemoresistance by modulating autophagy [34]. Fourth, there are genetic differences between right-sided and left-sided tumors, such as KRAS, BRAF, microsatellite instability (MSI), and TP53, and their prevalence are associated with sex [35, 36]. KRAS mutation and BRAF V600E mutation are associated with a poor prognosis, as they are predictive markers of resistance to epidermal growth factor-targeted antibodies [37, 38]. Furthermore, BRAF mutation was observed more frequently in females and proximal tumors [22]. MSI-high cancer is associated with the proximal colon and females, and is a predictive marker for fluoropyrimidine/oxaliplatin first-line chemotherapy [23]. Significantly, the rates of TP53 mutations increase as they are located distally [39], and TP53 mutation is more frequent in sporadic cancers among males than females [24]. Metastatic CRC patients with TP53 mutation who received chemotherapy had shorter survival [40]. These findings suggest that tumor-specific molecular changes are different among tumor locations as well as between the sexes.

Tumor-specific molecular changes and different biological molecular factors between males and females likely affect the chemotherapeutic effect. Sex differences contribute to innate and adaptive immunological responses, and several factors, including hormonal mediators, sex chromosomes, metabolic and bacterial mediators, and environmental mediators, influence the development process of the malignancies and therapeutic response of anticancer drugs [41]. A large population-based study demonstrated that females were an adverse independent predictor of survival in colon, rectal, and other cancers [42]. Another reason is that sex is related to differences in the toxicity of chemotherapeutic agents. Females are likely to experience severe toxicity with fluorouracil-based chemotherapy and oxaliplatin-based chemotherapy [43–45]. However, several recent large-scale studies demonstrated that females had significantly improved survival while experiencing more gastrointestinal toxicities in oesophagogastric cancer, lung cancer, and Hodgkin’s lymphoma [46–48].

These reports motivated us to investigate our hypothesis that the chemotherapeutic effects were affected by tumor location and sex. Our findings reveal that primary tumor sidedness is a promising prognostic indicator, regardless of sex, for unresectable colorectal cancer treated with chemotherapy. Our findings suggest that although sex differences may have contributed to treatment efficacy, the clinical implication was likely to be less than primary tumor-sidedness in clinical use. Further investigations into the associations between sex and molecular markers, including RAS, BRAF, and MSI, are needed.

This study has several limitations due to its retrospective nature. First, due to the 9-year study period, treatment strategies, such as chemotherapeutic regimens, have changed; thus, this study may not be fully reflective of current medical practice. Second, medical records about detailed treatment strategies, including chemotherapy regimen and dose and adverse events, were not obtained. Treatments for recurrence may have been based on the Japanese Society for Cancer of the Colon and Rectum (JSCCR) Guidelines 2005 for treatment of colorectal cancer [49]. First-line therapy may have been mostly L-OHP or CPT-11-based regimen; however, this study lacks these data, which may influence the results. Third, no data were available regarding molecular marker statuses, including RAS, BRAF, MSI, and TP53, which could potentially explain the prognostic differences between right-sided and left-sided tumors. Nevertheless, the present study is the first to demonstrate that tumor sidedness is an independent prognostic factor for unresectable recurrent colorectal cancer regardless of treatment strategy.

## Conclusions

Our findings demonstrated that right-sided tumors were associated with shorter cancer-specific survival than left-sided tumors; thus, tumor-sidedness should be considered a stratification parameter in further studies for recurrent unresectable CRC.

## Supplementary Information

**Additional file 1.**

## Data Availability

Data of the current study can be available from the corresponding author if requested.

## References

[1] Sung H, Ferlay J, Siegel RL (2021). Global Cancer statistics 2020: GLOBOCAN estimates of incidence and mortality worldwide for 36 cancers in 185 countries. CA Cancer J Clin.

[2] Matsuda K, Tamura K, Iwamoto H (2020). Tumor sidedness is associated with survival in patients with synchronous colorectal peritoneal carcinomatosis. Oncology.

[3] Zhang RX, Ma WJ, Gu YT (2017). Primary tumor location as a predictor of the benefit of palliative resection for colorectal cancer with unresectable metastasis. World J Surg Oncol.

[4] Shida D, Tanabe T, Boku N (2019). Prognostic value of primary tumor sidedness for unresectable stage IV colorectal cancer: a retrospective study. Ann Surg Oncol.

[5] Petrelli F, Tomasello G, Borgonovo K (2017). Prognostic survival associated with left-sided vs right-sided colon cancer: a systematic review and meta-analysis. JAMA Oncol.

[6] Ha GW, Kim JH, Lee MR (2019). Oncologic effects of primary tumor-sidedness on patients with stages 1-3 colon cancer: a Meta-analysis. Ann Surg Oncol.

[7] Karim S, Brennan K, Nanji S (2017). Association between prognosis and tumor laterality in early-stage colon cancer. JAMA Oncol.

[8] Warschkow R, Sulz MC, Marti L (2016). Better survival in right-sided versus left-sided stage I - III colon cancer patients. BMC Cancer.

[9] Ishihara S, Murono K, Sasaki K (2018). Impact of primary tumor location on postoperative recurrence and subsequent prognosis in nonmetastatic Colon cancers: a multicenter retrospective study using a propensity score analysis. Ann Surg.

[10] Malakorn S, Ouchi A, Hu CY (2021). Tumor sidedness, recurrence, and survival after curative resection of localized colon cancer. Clin Colorectal Cancer.

[11] Van Cutsem E, Cervantes A, Adam R (2016). ESMO consensus guidelines for the management of patients with metastatic colorectal cancer. Ann Oncol.

[12] Messersmith WA (2019). NCCN guidelines updates: Management of Metastatic Colorectal Cancer. J Natl Compr Cancer Netw.

[13] Hashiguchi Y, Muro K, Saito Y (2020). Japanese Society for Cancer of the Colon and Rectum (JSCCR) guidelines 2019 for the treatment of colorectal cancer. Int J Clin Oncol.

[14] Van Cutsem E, Köhne CH, Hitre E (2009). Cetuximab and chemotherapy as initial treatment for metastatic colorectal cancer. N Engl J Med.

[15] Douillard JY, Siena S, Cassidy J (2010). Randomized, phase III trial of panitumumab with infusional fluorouracil, leucovorin, and oxaliplatin (FOLFOX4) versus FOLFOX4 alone as first-line treatment in patients with previously untreated metastatic colorectal cancer: the PRIME study. J Clin Oncol.

[16] Schwartzberg LS, Rivera F, Karthaus M (2014). PEAK: a randomized, multicenter phase II study of panitumumab plus modified fluorouracil, leucovorin, and oxaliplatin (mFOLFOX6) or bevacizumab plus mFOLFOX6 in patients with previously untreated, unresectable, wild-type KRAS exon 2 metastatic colorectal cancer. J Clin Oncol.

[17] Heinemann V, von Weikersthal LF, Decker T (2014). FOLFIRI plus cetuximab versus FOLFIRI plus bevacizumab as first-line treatment for patients with metastatic colorectal cancer (FIRE-3): a randomised, open-label, phase 3 trial. Lancet Oncol.

[18] Stintzing S, Modest DP, Rossius L (2016). FOLFIRI plus cetuximab versus FOLFIRI plus bevacizumab for metastatic colorectal cancer (FIRE-3): a post-hoc analysis of tumour dynamics in the final RAS wild-type subgroup of this randomised open-label phase 3 trial. Lancet Oncol.

[19] CALGB/SWOG (2006). C80405: a phase III trial of FOLFIRI or FOLFOX with bevacizumab or cetuximab or both for untreated metastatic adenocarcinoma of the colon or rectum. Clin Adv Hematol Oncol.

[20] Peeters M, Price TJ, Cervantes A (2010). Randomized phase III study of panitumumab with fluorouracil, leucovorin, and irinotecan (FOLFIRI) compared with FOLFIRI alone as second-line treatment in patients with metastatic colorectal cancer. J Clin Oncol.

[21] Arnold D, Lueza B, Douillard JY (2017). Prognostic and predictive value of primary tumour side in patients with RAS wild-type metastatic colorectal cancer treated with chemotherapy and EGFR directed antibodies in six randomized trials. Ann Oncol.

[22] Kalady MF, Dejulius KL, Sanchez JA (2012). BRAF mutations in colorectal cancer are associated with distinct clinical characteristics and worse prognosis. Dis Colon Rectum.

[23] Liu J, Wang B, Fang W (2020). Microsatellite instability and sensitivity to fluoropyrimidine and oxaliplatin containing first-line chemotherapy in metastatic colorectal cancer. Eur J Hosp Pharm.

[24] Haupt S, Caramia F, Herschtal A (2019). Identification of cancer sex-disparity in the functional integrity of p53 and its X chromosome network. Nat Commun.

[25] Kaiser AM, Kang JC, Chan LS (2006). The prognostic impact of the time interval to recurrence for the mortality in recurrent colorectal cancer. Color Dis.

[26] Westberg K, Palmer G, Johansson H (2015). Time to local recurrence as a prognostic factor in patients with rectal cancer. Eur J Surg Oncol.

[27] Li H, Jin X, Liu P (2017). Time to local recurrence as a predictor of survival in unrecetable gastric cancer patients after radical gastrectomy. Oncotarget.

[28] Shida D, Inoue M, Tanabe T (2020). Prognostic impact of primary tumor location in stage III colorectal cancer-right-sided colon versus left-sided colon versus rectum: a nationwide multicenter retrospective study. J Gastroenterol.

[29] Lee JM, Han YD, Cho MS (2019). Impact of tumor sidedness on survival and recurrence patterns in colon cancer patients. Ann Surg Treat Res.

[30] Sparreboom CL, van Groningen JT, Lingsma HF (2018). Different risk factors for early and late colorectal anastomotic leakage in a Nationwide audit. Dis Colon Rectum.

[31] Riihimäki M, Hemminki A, Sundquist J (2016). Patterns of metastasis in colon and rectal cancer. Sci Rep.

[32] Meltzer S, Bakke KM, Rød KL (2020). Sex-related differences in primary metastatic site in rectal cancer; associated with hemodynamic factors?. Clin Transl Radiat Oncol.

[33] Gao Z, Guo B, Gao R (2015). Microbiota disbiosis is associated with colorectal cancer. Front Microbiol.

[34] Yu T, Guo F, Yu Y (2017). Fusobacterium nucleatum promotes Chemoresistance to colorectal Cancer by modulating autophagy. Cell.

[35] Missiaglia E, Jacobs B, D'Ario G (2014). Distal and proximal colon cancers differ in terms of molecular, pathological, and clinical features. Ann Oncol.

[36] Guinney J, Dienstmann R, Wang X (2015). The consensus molecular subtypes of colorectal cancer. Nat Med.

[37] Allegra CJ, Jessup JM, Somerfield MR (2009). American Society of Clinical Oncology provisional clinical opinion: testing for KRAS gene mutations in patients with metastatic colorectal carcinoma to predict response to anti-epidermal growth factor receptor monoclonal antibody therapy. J Clin Oncol.

[38] Roth AD, Tejpar S, Delorenzi M (2010). Prognostic role of KRAS and BRAF in stage II and III resected colon cancer: results of the translational study on the PETACC-3, EORTC 40993, SAKK 60-00 trial. J Clin Oncol.

[39] Loree JM, Pereira AAL, Lam M (2018). Classifying colorectal Cancer by tumor location rather than sidedness highlights a continuum in mutation profiles and consensus molecular subtypes. Clin Cancer Res.

[40] Benhattar J, Cerottini JP, Saraga E (1996). p53 mutations as a possible predictor of response to chemotherapy in metastatic colorectal carcinomas. Int J Cancer.

[41] Klein SL, Flanagan KL (2016). Sex differences in immune responses. Nat Rev Immunol.

[42] Radkiewicz C, Johansson ALV, Dickman PW (2017). Sex differences in cancer risk and survival: a Swedish cohort study. Eur J Cancer.

[43] Sloan JA, Loprinzi CL, Novotny PJ (2000). Sex differences in fluorouracil-induced stomatitis. J Clin Oncol.

[44] Nozawa H, Kawai K, Sasaki K, et al. Women are predisposed to early dose-limiting toxicities during adjuvant CAPOX for colorectal cancer. Int J Clin Pract. 2021;75(11):e14863. 10.1111/ijcp.14863 34516723

[45] Yamada Y, Koizumi W, Nishikawa K (2019). Sex differences in the safety of S-1 plus oxaliplatin and S-1 plus cisplatin for patients with metastatic gastric cancer. Cancer Sci.

[46] Athauda A, Nankivell M, Langley RE (2020). Impact of sex and age on chemotherapy efficacy, toxicity and survival in localised oesophagogastric cancer: a pooled analysis of 3265 individual patient data from four large randomised trials (OE02, OE05, MAGIC and ST03). Eur J Cancer.

[47] Singh S, Parulekar W, Murray N (2005). Influence of sex on toxicity and treatment outcome in small-cell lung cancer. J Clin Oncol.

[48] Klimm B, Reineke T, Haverkamp H (2005). Role of hematotoxicity and sex in patients with Hodgkin's lymphoma: an analysis from the German Hodgkin study group. J Clin Oncol.

[49] Japanese Society for Cancer of the Colon and Rectum (2005). JSCCR guidelines 2005 for the treatment of colorectal Cancer.

